# From Biological Cilia to Artificial Flow Sensors: Biomimetic Soft Polymer Nanosensors with High Sensing Performance

**DOI:** 10.1038/srep32955

**Published:** 2016-09-13

**Authors:** Mohsen Asadnia, Ajay Giri Prakash Kottapalli, K. Domenica Karavitaki, Majid Ebrahimi Warkiani, Jianmin Miao, David P. Corey, Michael Triantafyllou

**Affiliations:** 1 School of Mechanical & Aerospace Engineering, Nanyang Technological University, 50 Nanyang Avenue, 639798 Singapore; 2 Department of Engineering, Macquarie University, Sydney, New South Wales 2109, Australia; 3 Center for Environmental Sensing and Modeling (CENSAM) IRG Singapore-MIT Alliance for Research and Technology (SMART) Centre, 3 Science Drive 2, 117543 Singapore; 4 Howard Hughes Medical Institute and Department of Neurobiology, Harvard Medical School, 220 Longwood Avenue, Boston, MA 02115, USA; 5 School of Mechanical and Manufacturing Engineering, Australian Centre for NanoMedicine, University of New South Wales, Sydney, New South Wales 2052, Australia; 6 Department of Mechanical Engineering, Massachusetts Institute of Technology, 77 Massachusetts Avenue, Cambridge, MA 02139, USA

## Abstract

We report the development of a new class of miniature all-polymer flow sensors that closely mimic the intricate morphology of the mechanosensory ciliary bundles in biological hair cells. An artificial ciliary bundle is achieved by fabricating bundled polydimethylsiloxane (PDMS) micro-pillars with graded heights and electrospinning polyvinylidenefluoride (PVDF) piezoelectric nanofiber tip links. The piezoelectric nature of a single nanofiber tip link is confirmed by X-ray diffraction (XRD) and Fourier transform infrared spectroscopy (FTIR). Rheology and nanoindentation experiments are used to ensure that the viscous properties of the hyaluronic acid (HA)-based hydrogel are close to the biological cupula. A dome-shaped HA hydrogel cupula that encapsulates the artificial hair cell bundle is formed through precision drop-casting and swelling processes. Fluid drag force actuates the hydrogel cupula and deflects the micro-pillar bundle, stretching the nanofibers and generating electric charges. Functioning with principles analogous to the hair bundles, the sensors achieve a sensitivity and threshold detection limit of 300 mV/(m/s) and 8 μm/s, respectively. These self-powered, sensitive, flexible, biocompatibale and miniaturized sensors can find extensive applications in navigation and maneuvering of underwater robots, artificial hearing systems, biomedical and microfluidic devices.

Many animal sensory systems use mechanotransduction which is the conversion of a mechanical stimulus to an electrical signal. Mechanosensitive cells and tissues employ a diverse set of exceptionally sensitive sensors to detect various signals including pressure, touch, sound, acceleration and fluid flow^1^,^2^,^3^,^4^,^5^. Nature’s evolutionary path led to sensors of high functionality and robustness, in terms of material properties, anatomical architecture and energy expenditure. In the vertebrate inner ear, ultrafast and sub-Brownian threshold detection of sound, linear acceleration and angular velocity is accomplished by mechanosensitive cells that exhibit microsecond response times and nanometer-scale deflection sensitivities^6^. These cells are called hair cells from the appearance of their sensing structures—micrometer-scale bundles of actin-based stereocilia, called hair bundles that protrude from their apical surfaces. Hair cells are also found in the fish lateral line system where they sense the velocity and direction of water flow. The velocity sensitivity threshold for flow receptors in fish was estimated, using a dipole stimulus, to be 18–38 μm/s for the frequency range of 10–20 Hz^7^,^8^ and 1 cm/s for steady-state flows^9^. The ubiquity in vertebrates and the outstanding sensing performance of hair cells qualify them as prime biomimetic candidates to engineer artificial flow sensors. Here we present a new class of miniaturized, biocompatible, self-powered and flexible microelectromechanical system (MEMS) flow sensors that achieve high voltage sensitivity and low velocity detection thresholds by closely mimicking the anatomy and function of hair cells.

Hair cells of the mammalian auditory and vestibular systems and hair cells of many non-mammalian vertebrates (e.g., bullfrog; Fig. 1a) exhibit structural and functional similarity. In all these systems, hair cells respond to deflection of their hair bundles. Different bundles consist of 30–300 stereocilia from 1 μm to over 60 μm tall, and usually a single microtubule-based kinocilium, and always organized in a formation of rows with step-wise increasing heights from the shortest to the longest (closest to the kinocilium)^10^,^11^ (Fig. 1a–d). The tips of all the stereocilia are connected with fine filaments called “tip links”^12^, which connect the graded height columns with each link extending upward connecting to its tallest neighbour (Fig. 1b–d). External stimuli deflect the ciliary bundle as a unit by a mechanism of sliding adhesion: stereocilia pivot at their bases without appreciable bending along their shafts and adjacent stereocilia stick together laterally but still shear while they pivot (Fig. 1b)^13^. The increasing heights of the stereocilia confer a direction-dependent axis of stimulus excitation: stimuli toward the tallest stereocilia (excitatory x-direction; “+” in Fig. 1b) increase tension in the tip links and open force-gated ion channels (Fig. 1d); stimuli toward the shortest stereocilia (inhibitory x-direction; “−” in Fig. 1b) relax the tip links and allow channels to close^14^,^15^. The directional response of the cell is therefore maximum when the stimulus deflects the bundle in the excitatory x-direction and decreases approximately as the cosine of the angle by which the stimulus direction differs from the x-direction, to zero for stimuli perpendicular to the x-direction^16^,^17^. Ion channel opening results in inward ionic current flow, depolarization of the cell body, neurotransmitter release and finally the initiation of action potentials in the afferent nerve fibers connecting the sensory periphery to the brain^18^,^19^.

The fish lateral line system consists of an array of superficial neuromast sensors 10–500 μm in diameter and 100–1000 μm tall, each composed of two sets of hair cells arranged with their hair bundles oriented oppositely^20^,^21^. The stereocilia and long kinocilia of all cells in a neuromast are embedded in a common gelatinous cupula that mechanically couples external flows to the stereocilia^20^,^22^. Therefore, flow in a particular direction excites one set of hair cells and inhibits the oppositely-oriented cells; the differential comparison enhances the directional sensitivity of the neuromast^23^.

The sensing abilities achieved by biological hair cell sensors are beyond the capabilities of most human-engineered sensors available today. Design of hair cell-like sensors for artificial MEMS flow sensors can offer several advantages over existing devices. It can imitate novel structural and functional principles that are already proven to work in their native biological setting. Amalgamating this biomimetic advantage with the mature micro/nanofabrication technologies will enable the development of devices with sensing abilities that surpass those of conventionally engineered sensors and match the high sensitivity of their biological counterparts.

In most of the hair cell inspired flow sensors developed in the past, flow-generated deflection was transduced into an electrical output utilizing piezoresistive and piezoelectric sensing elements embedded in a MEMS cantilever or membrane^24^,^25^,^26^,^27^,^28^,^29^,^30^,^31^,^32^,^33^. Engineering a replication of the intricate morphological organization of the hair cell is a complex and challenging task. Therefore, in all the MEMS sensor developments, the basic MEMS sensor design followed a bio-inspired approach wherein; the ciliary bundle was approximated as a single cylindrical pillar deflected by flow^24^,^25^,^28^,^30^,^31^,^32^,^33^. Chen N. *et al*. developed an artificial haircell sensor consisting of a silicon cantilever beam embedded with piezoresistive sensing elements at the hinge and a high-aspect-ratio cilium attached at the distal end^32^. They demonstrated a threshold detection limit of 0.7 mm/s for sensing oscillatory flows in water generated using a dipole stimulus. They also achieved a high angular flow resolution of 2.16° in detecting the sensor’s yaw response in air. Yang Y. *et al*. developed arrays of flow sensors which function based on the principle of thermal hot wire anemometry and are capable of underwater localisation of a dipole source^28^. Asadnia M. *et al*. developed self-powered MEMS hair cell sensors that featured piezoelectric membranes at the base of the long hair cells that interact with flow^34^. In more recent works, innovative ideas in designing neuromast inspired flow sensors have been proposed through synthesis of artificial cupula-like hydrogel materials^24^,^30^,^31^,^33^. Researchers also incorporated a cupula-like encapsulation over the hair cells through drop-casting techniques^24^,^30^,^31^,^33^. The biomimetic hydrogel cupula has been demonstrated to enhance the sensing performance of the flow sensors by maximizing and mediating drag forces along the haircell and thereby improving the sensitivity and accuracy in flow sensing^24^,^30^,^31^,^33^.

Here we present a novel, completely biomimetic flow sensor which attempts to replicate the intricate morphological organization and function of the hair bundles of the hair cells within the fish neuromasts. The nanofiber links which form the key sensing elements do not reside at the substrate plane (i.e., at the bottom of the boundary layer) but rather at the tips of the pillar bundle in a geometry similar to the tip links in the biological hair bundle. We fabricated graded rows of flexible, bundled, polydimethylsiloxane (PDMS) polymer pillars to form a structure similar to a hair bundle. Flow direction sensing in water is not achieved by most of the existing artificial flow sensors. The biomimetic design of the graded pillars, enables flow velocity sensing and flow direction sensing functionality. Electrospun piezoelectric polyvinylidene fluoride (PVDF) polymer nanofibers connect consecutive tips of the pillars to mimic the biological tip links. Hyaluronic acid (HA)-based hydrogels, closely matching the material properties of the biological cupula, were synthesized and drop-cast on the stereocilia-like pillars to form artificial cupulas. These serve as a mechanical connection between the external flow and the pillars and enhance sensitivity by increasing drag force. Finally, unlike the silicon-based flow sensors developed in the past^25^,^27^,^28^,^31^,^32^,^33^,^34^, our all-polymer devices are biocompatible, self-powered and inexpensive.

## Results and Discussions

### Structural design of the artificial MEMS ciliary bundle

**Figure 2:**
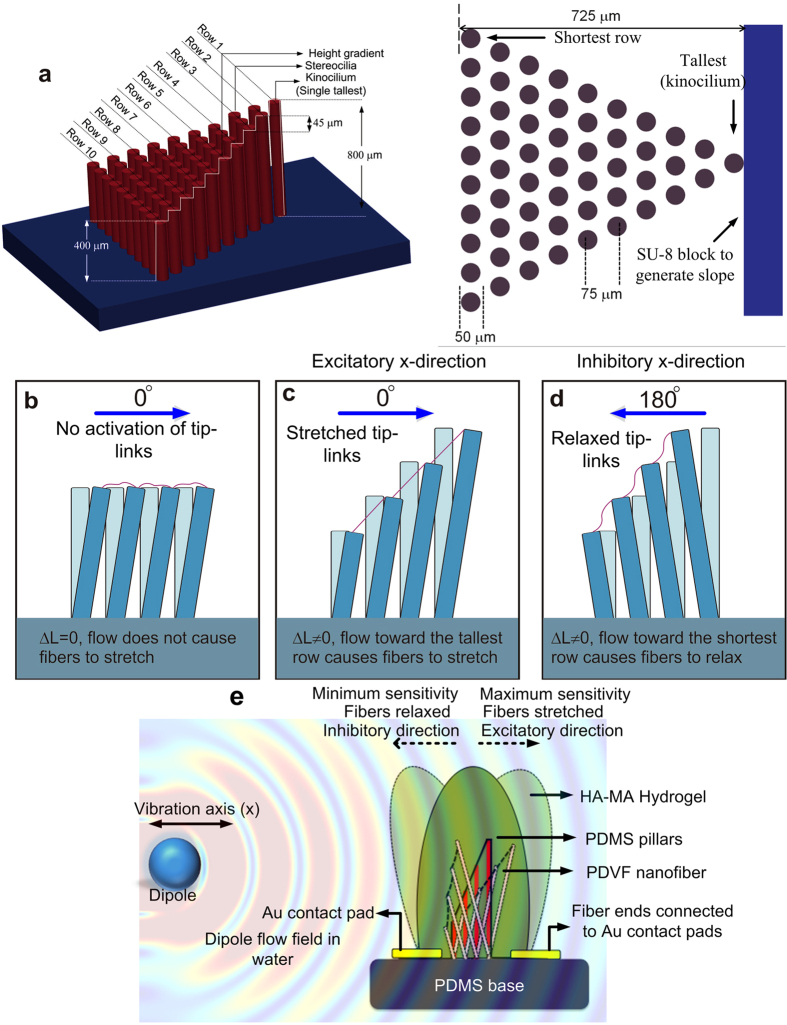
Design and working mechanism of the biomimetic MEMS artificial hair cell sensor. (**a**) Angled side-view and top-view of the graded height and geometrical dimensions of PDMS pillars within a MEMS bundle. (**b–d**) Schematics illustrating the necessity of height gradation in the design of pillar bundle and the directional dependence of the output, for different directions of flow along the bundle’s axis. (**e**) A schematic showing the flow sensing mechanism in the presence of an oscillating dipole.

The MEMS artificial hair cell sensor features a bundle of high-aspect-ratio PDMS pillars with a geometrical arrangement analogous to the biological hair cell bundle. Each bundle consists of a single tallest pillar (in the position of the kinocilium) and 54 shorter pillars (analogous to stereocilia) arranged in 10 rows (Fig. 2a). The heights of the pillars increase monotonically in steps of 45 μm along a column from one edge of the bundle to the other but are equal within a row. The graded height of the PDMS pillars ranges from 400 μm for the shortest pillar row to 800 μm for the kinocilium as shown in Fig. 2a. Each taller pillar of a successive row falls at the centre of the two pillars of the preceding shorter row leading to a hexagonal symmetry in the overall pillar geometry within a single hair cell bundle. This arrangement maximizes the number of tip links generated between the pillars and also ensures that all the tip links have similar length, equal to the distance between the rows. Each PDMS pillar has a diameter of 50 μm and adjacent pillar rows are spaced 75 μm apart (measured center-to-center). The distance between the tallest and the shortest pillar is 725 μm. The design of the pillars allows the detection of flow velocity and flow direction analogous to the mechanotransduction principle in hair cells. The design also takes into account other parameters such as feasibility of microfabrication to achieve such height gradient in the pillars (see Supplementary Information S1), and the effect of boundary layers generated due to flow-structure interaction on the sensitivity of the sensor.

The tip links in the artificial stereocilia sensor are formed through electrospinning piezoelectric PVDF nanofibers. PVDF has been chosen as the sensing element due to its highest piezoelectric coefficient among polymer materials, biocompatibility and its flexibility which allows formation of nanofibers through electrospinning process^35^. PVDF forms in different crystal structures depending on sample preparation conditions. In general, the α-phase of PVDF is the most common structure, typically obtained when the PVDF is cooled and solidified from melt. While the α-phase is a non-polar structure that does not show piezoelectricity, the β-phase has ferroelectric crystalline structure with a strong piezoelectric effect. In general, high mechanical (approximately 50%) and electrical stretching is required to convert most of the PVDF from α-phase to β-phase. In this study, in order to confirm the occurrence of the β-phase, the molecular and crystalline structure of the electrospun PVDF was studied by XRD and FTIR (see Supplementary Information S2).

An important component of the sensor which enhances its sensitivity as well as acts as a package protecting the nanofiber tip links and the PDMS stereocilia is the cupula. Hyaluronic acid hydrogel encapsulates the PDMS pillars and extends nearly to a height twice of the tallest PDMS pillar. The hydrogel cupula captures and transmits the fluid drag force to the embedded stereocilia. The presence of the hydrogel cupula enhances the sensitivity of the MEMS flow sensor in many ways. The height and diameter of the hydrogel cupula are larger than those of the hair cells; hence increased surface area projected to the flow leads to an increased drag force and thereby an enhanced sensitivity. In addition, the increased height of the cupula over the hair bundles causes the structure to extend beyond the boundary layer, further enhancing sensitivity. Hydrogel is a highly porous material and there exists a coupling interaction between the fluid trapped inside the loose semi-permeable hydrogel pores and the external flow which further improving the sensitivity of the sensor^31^,^33^,^36^. Hydrogel binds the PDMS pillars together allowing them to stick together laterally and depict a cohesive displacement in the presence of flow stimulus similar to the biological stereocilia^13^,^37^.

### Sensing principle

The sensor operates through the deflection of the artificial cupula in response to flow disturbances, resulting in the bending of the embedded PDMS pillars. The graded height of the pillars confers flow sensing and directional dependence. If all pillars were the same height (Fig. 2b), stimuli would equally displace all rows within the bundle and the interconnecting PVDF nanofibers would not stretch. With pillars arranged in rows of graded height, excitatory stimuli (Fig. 2c) cause a differential displacement at the tip of adjacent pillars and the PVDF nanofibers stretch. Conversely, inhibitory stimuli cause the fibers to relax (Fig. 2d). These nanofibers are the principle sensing elements of the MEMS flow sensor. When a tensile stress is applied to the PVDF nanofibers it induces piezoelectric charges which produce a potential difference along the polling direction across the contact pads (Fig. 2e). When the pillars relax back, the piezoelectric potential between the two contacts decreases and the locally accumulated free carriers at both ends of the fibers generate an opposing potential. The charges collected at both ends through contact pads generate the sensor’s voltage output. This design also allows a directional dependent voltage output of the sensor. The sensor exhibits a maximum and minimum sensitivity when the orientation of flow with respect to the pillar array is at 0° (Fig. 2c) and 180° (Fig. 2d) respectively.

### Sensor fabrication

The fabrication of the artificial stereocilia flow sensor combines soft-polymer synthesis methods and nanofiber generation techniques with conventional microfabrication methods to replicate the morphology of a biological hair bundle. The device fabrication consists of three major steps: (1) fabrication of pillars in rows of graded heights; (2) formation of electrospun PVDF nanofiber links; and (3) development of a HA-MA hydrogel cupula.

**Figure 3:**
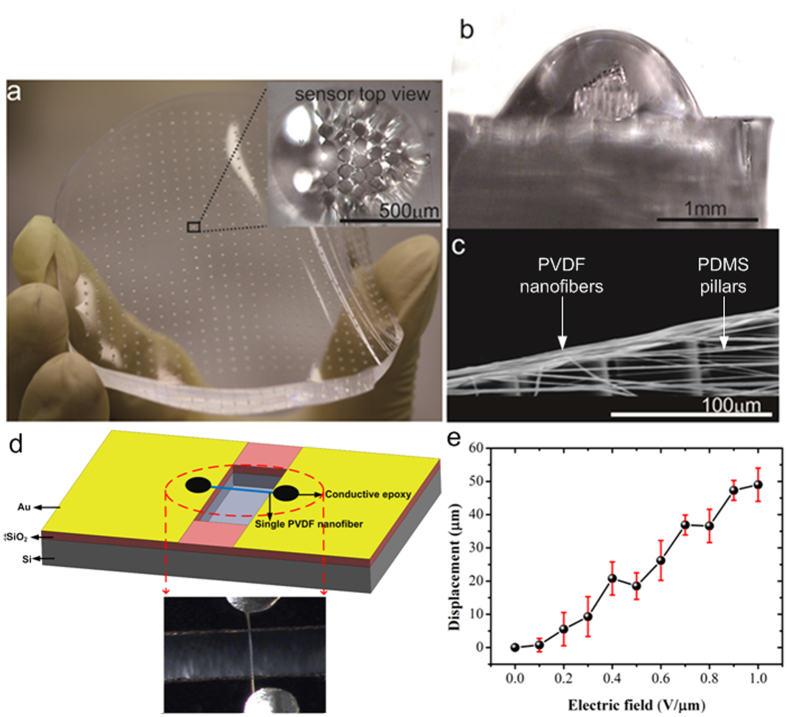
Fabrication of MEMS hair cell sensors. (**a**) A flexible PDMS wafer with a number of PDMS pillar bundles. Insert shows the top view of the sensor through the hydrogel cupula. The tips of the pillars can be seen through the transparent hydrogel. (**b**) Microscopic side-view image of the sensor showing the hydrogel cupula and the PDMS pillars with height gradient. Note that the row of shortest pillars is closest to the viewer. (**c**) SEM image showing the tips of four pillars and the tip links connecting them. (**d**) Schematic and optical image of the MEMS device fabricated to determine the piezoelectric coefficient of a single nanofiber. The device consists of a single nanofiber suspended on a DRIE cavity in silicon and bridges two gold contact pads. (**e**) Nanomechanical electrical characterization of a single PVDF nanofiber to determine the piezoelectric coefficient of the fiber. Error bars are S.D.; n = 5.

It is challenging to fabricate a bundle of closely spaced pillars of the same diameter but varying heights using standard microfabrication methods. A bundle of flexible pillars was achieved by initially fabricating the micropillars using SU-8 and transferring the same pattern to PDMS through a double moulding process. SU-8 pillars are hard and brittle, limiting the sensitivity and reliability of the sensor. One of the critical step in the fabrication of micro-pillars with a height gradient was the SU-8 chemical development process. The desired gradient was obtained by lithographically patterning SU-8 through a partial development process that exploits the low development rates of SU-8 at the convex corners under no-agitation conditions. The development process was followed by a thermal reflow process. To obtain the desired gradient, optimization of the development and exposure times was required during SU-8 patterning. Figure 3a shows a flexible PDMS substrate featuring a number of artificial hair bundles. A magnified top view of one of these bundles (inset in Fig. 3a) identifies individual pillars; a side view (Fig. 3b) highlights the graded heights of these pillars and the transparent surrounding cupula. The nanofabrication of the artificial hair bundle with complete processing details is explained in Supplementary Information S1.

We developed artificial PVDF nanofiber tip links to connect the tips of the pillars through an electrospinning process (see Supplementary Information S2 and S3). Figure 3c is a scanning electron micrograph showing the tips of four pillars and their interconnecting nanofibers. Before the placement of nanofibers on the pillars, we conducted a complete electrical and mechnical characterization of the nanofibers. A single nanofiber suspended between two gold contacts pads was developed by electrospinning PVDF on silicon substrates featuring a cavity formed by deep reactive ion etching (DRIE) (Fig. 3d). Nanomechanical characterizations conducted using a TriboScan 950 triboindenter (Hysitron, MN, USA) revealed a Young’s modulus and hardness of 2.2 GPa and 0.1 GPa, respectively. Electrical characterization of a single nanofiber was conducted to investigate the piezoelectric coefficient of the nanofiber. By applying electrical fields ranging from 0 to 1 V/mm across the electrodes, the maximum deformation of fibers at the center point was measured by imaging the fibers under a confocal microscope (PLμ Sensorfar confocal imaging profiler). Figure 3e shows the displacements of the nanofibers observed under the confocal microscope for various electric fields applied. The results demonstrate an increase in the displacement of the nanofiber with increasing electric field, and a high piezoelectric coefficient of d_33_ = −58.7 pm/V for a single PVDF nanofiber.

**Figure 4:**
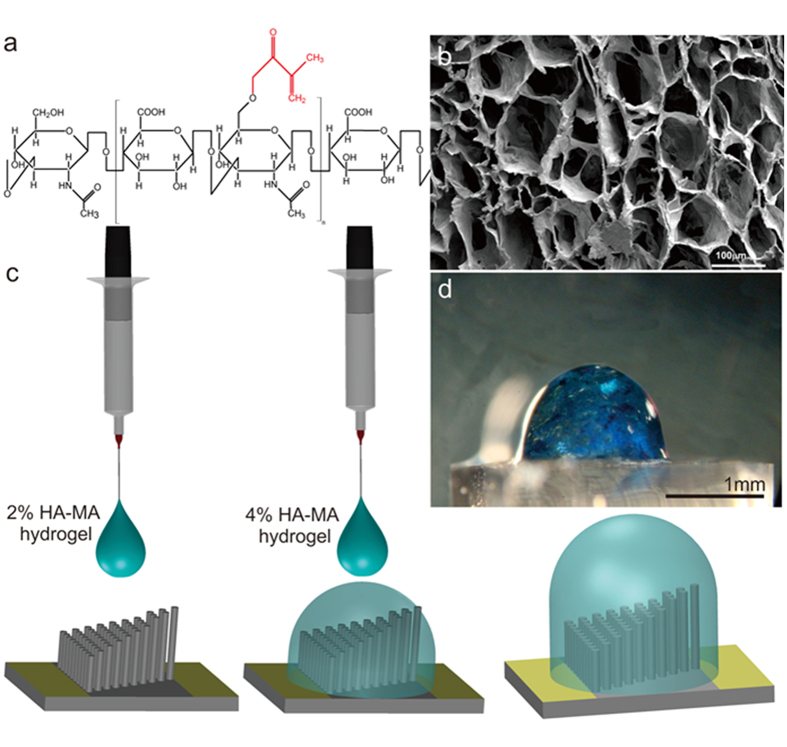
HA-MA Hydrogel cupula. (**a**) Chemical structure of HA-MA macromer. The attached chemical moiety is marked in red. (**b**) Porous structural organization of HA-MA hydrogel revealed through SEM images of cross-section samples of hydrogel. (Photo courtesy of Meghali Bora, Department of Material Science and Engineering, Nanyang Technological University, Singapore). (**c**) A schematic of the drop-casting process. (**d**) A side-view optical microscope image of the MEMS sensor after the cupula drop-casting. The hydrogel cupula is treated with methylene blue dye.

The Young’s modulus of the biological cupula (the superficial neuromast in zebra fish) is reported to be 10–100 Pa^38^. To develop an artificial cupula, we identified hyaluronic acid-methacrylic anhydride (HA-MA) hydrogel (Fig. 4a) as the most suitable material, with the closest properties to the biological cupula. Solutions of 2% and 4% HA-MA hydrogels (Fig. 4b) mixed with 0.1% Irgacure 2959 initiator were used to form the cupula. We conducted nanoindentation analysis and rheological characterizations to determine the material properties such as the Young’s modulus and complex viscosity of the hydrogels using an Agilent Technologies G200 nano-indenter and an Anton-Parr MCR501 stress-controlled rheometer, respectively. Details of the hydrogel material characterization are in Supplementary Information S4. HA-MA hydrogel has a density close to the density of water and therefore the cupula is mainly driven through viscous forces^23^. The biological cupula not only enhances the sensitivity of the hair-cell sensors, but also diminishes the effects of high frequency flows and Brownian motion, thereby increasing the signal-to-noise ratio of the neuromast sensors^39^. The hydrophilicity and permeability of the hydrogel material increase the signal absorption through an enhanced friction factor associated with the material^30^,^31^. The hydrogel cupula was formed on the PDMS stereocilia through a controlled drop-casting method (Fig. 4c). Drop-casting was performed in a two-step process designed to ensure that the hydrogel cupula had a high aspect ratio and did not spread over onto the contact pads (Fig. 4d). Initially a 2% hydrogel solution was drop-cast followed by a 4% hydrogel solution. The 2% hydrogel solution was less viscous and flowed through the nanofibers and the gaps between the pillars thus binding them together. The 4% hydrogel is more viscous and enabled an increase in height with minimal spread to the contact pads, producing a cupula 1500 μm tall and 1000 μm in diameter. Details on the drop-casting method are in Supplementary Information S4.

Peleshenko. *et al*.^33^ developed a simple analysis to theoretically estimate the sensitivity enhancement due to the presence of the cupula over the naked hair cells. At low Reynolds numbers, the drag force F exerted on a prolate-shaped cupula is F = C·μ·L·U, where C is a constant, U is the flow velocity, L is the characteristic length or the diameter of the structure facing the flow, and μ is the dynamic viscosity. Using a simple scaling analysis, the ratio of the drag force exerted on a hydrogel cupula as compared to the naked hair cell sensors can be estimated using a prolate shape approximation, as follows


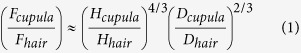


Using the dimensions of the cupula and the hair cell of the MEMS sensor, the drag force due to the presence of the cupula is 4.2 times higher than for the naked stereocilia. The height of the hair cell was taken to be the average height of all the 55 stereocilia (600 μm) and the diameter of the hair cell was taken to be the distance between the shortest hair cell row and the longest kinocilium (725 μm). In reality, the enhancement in sensitivity due to the presence of the cupula would be higher than 4.2 since the formula only considers the mechanical contribution of the hydrogel cupula in terms of the drag force enhancement, but does not consider the contribution to sensitivity enhancement due to hydrogel’s material characteristics.

### Flow sensing experiments

**Figure 5:**
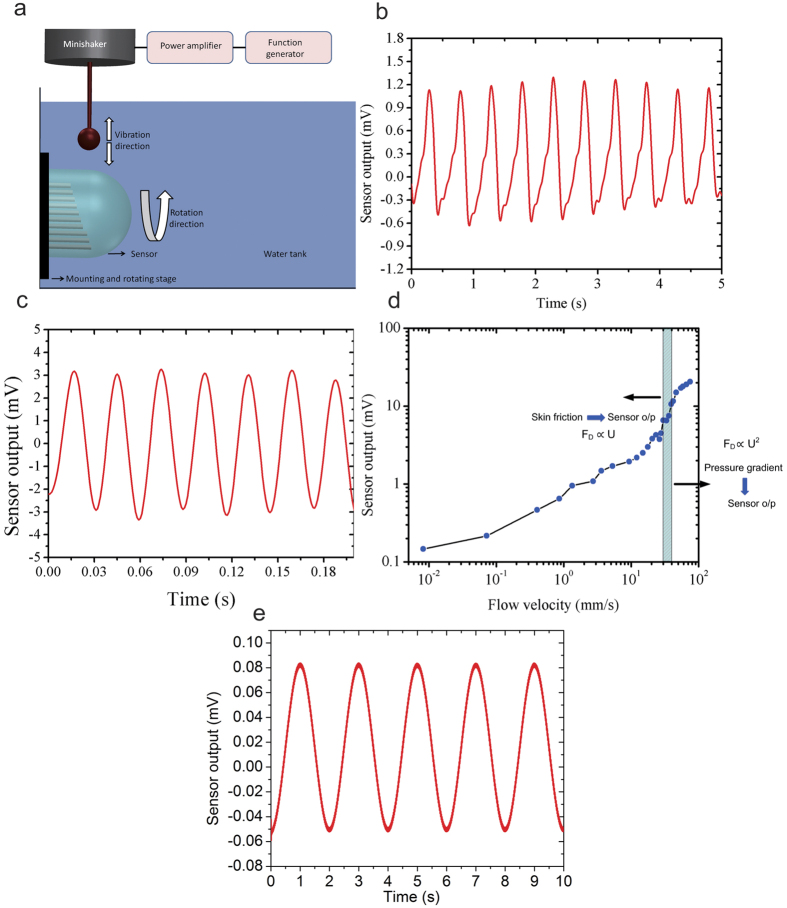
Flow sensing experiments. (**a**) Schematic showing the basic experimental setup used in all the experiments. (**b**) The sensor output follows the vibration frequency of the dipole at 2 Hz, and (**c**) at 35 Hz. (**d**) Sensitivity and threshold-detection limit of the sensors over a range of velocities. Pink bar indicates the velocity range for a transition from skin friction to pressure gradient as the major determinant of drag force. (**e**) Time-dependent sensor output at threshold detection limit of 8 μm/s.

The flow sensing performance of the MEMS artificial hair cell sensor was investigated by recording the response of the sensor to a dipole (vibrating sphere) stimulus in water. A dipole stimulus was used to characterize the flow sensing abilities of the sensor for three reasons. First, the theory of dipole flow fields has been well studied in the past^40^,^41^, which allows simple analytical validations of experimental results. Second, the dipole-generated flow fields closely represent those generated by swimming fish^41^,^42^. Third, most biologists and engineers who developed bioinspired sensors inspired by the functionality of the lateral line system used a dipole stimulus, which allows a comparison of the sensing performance of the sensors. A sphere of 8-mm diameter was driven with sinusoidal signals of a fixed amplitude of 250 mV_rms_ and frequencies (0–100 Hz) to generate oscillatory flows (Fig. 5a). Prior to sensor testing, the dipole source was calibrated using a laser Doppler vibrometer (LDV) to determine the sphere velocity when driven at various frequencies^34^. Figure 5a schematically depicts the experimental setup used in all the three experiments. The dipole was positioned 25 mm away from the sensor and was vibrated in a direction perpendicular to the long axis of the pillars. The voltage output from the sensor was amplified 500X using a low-noise preamplifier, digitized, and recorded in LabVIEW.

In one experiment, the dipole was driven with a sinusoidal signal of 0.5 Hz, 2 Hz and 35 Hz and amplitude of 250 mV_rms_. The vibration of the dipole causes the water in the vicinity of the dipole to move at the same frequency, forcing the cupula to bend, bending the pillars as described above, and thus generating electric charge. Figure 5b,c show the output of the sensor to a dipole vibrating at 2 Hz and 35 Hz. It can be observed that the sensor output clearly follows the frequency of the stimulus, with a minor deviation from ideal sinusoidal wave pattern at the frequency of 2 Hz. This is attributed to the frequency range of the minishaker that drives the dipole. Below the frequency of 20 Hz, although the minishaker does respond accurately to the driving frequency, the sinusoidal displacement pattern of the membrane of the minishaker is distorted.

To determine the voltage sensitivity and velocity-detection threshold of the flow sensor, varying flow velocities from 1 μm/s to 80 mm/s were generated by adjusting the frequency of the sinusoidal signal fed to the dipole. The flow velocity generated by the dipole at the location of the sensor is a function of sphere diameter, angular vibration frequency, frequency of vibration, displacement amplitude of the dipole and the distance between the center of the dipole and the sensors (observation distance)^34^,^42^. The flow velocity at the location of the sensor was derived through LDV calibrations of the dipole and flow field models of a vibrating sphere in fluid^34^.

Figure 5d shows the flow velocity calibration of the sensor. The results presented are average results by performing the same experiment on 5 sensors. The results are plotted in logarithmic scale to show the threshold detection limit clearly. The sensor’s output varied monotonically with the amplitude of the sinusoidal source, as expected. The sensors demonstrated a velocity threshold of 8 μm/s. Figure 5e shows the time-dependent sensor output for the case of threshold flow velocity of 8 μm/s, which corresponds to a dipole stimulus frequency of 0.5 Hz and amplitude of 250 mV_rms_. The voltage sensitivity of the sensors calculated from the flow calibration experiments was 300 mV/(m/s). The sensitivity and threshold-detection limit achieved by our sensors are higher than for many flow sensors developed in the past and are on a par with biological sensors^7^,^8^,^9^. For instance, receptors in fish show a velocity detection threshold of 18–38 μm/s in the 10–20 Hz frequency range for a dipole stimulus^7^,^8^,^9^. However, there is a transition in slope at flow velocities of 30–40 mm/s. This is not due to the response of the sensor to flow, but mostly due to the mechanics of flow-structure interaction. The Reynolds number (R_e_) for flows at this transition point is 50–70. Skin friction dominates in contribution to sensor output at flow velocities below this R_e_. However, at higher velocities beyond 30–40 mm/s, the contribution of pressure gradient dominates. While drag force due to skin friction is linearly proportional to flow velocity, drag force due to pressure gradient is quadratically proportional to velocity, which causes a steeper increase in sensor output at velocities beyond 30–40 mm/s.

**Figure 6:**
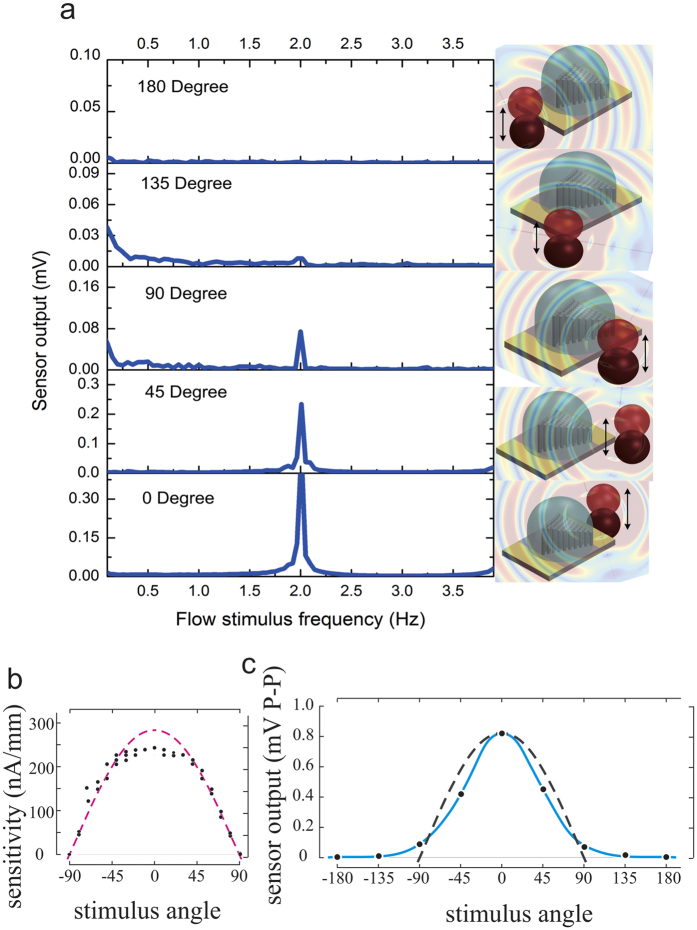
Experimental results showing the similarity in the direction dependant output of the MEMS hair cell and the biological hair cell in the bullfrog sacculus. (**a**) The amplitude of the sensor output at the stimulus frequency (2 Hz) for various orientations of the flow (black dotted line) with respect to the axis of the hair bundle (black solid line). Schematic annotations describing 0°, 45°, 90°, 135°, 180° orientations are shown beside the corresponding plots. (**b**) Experimental measurements (filled circles) showing the ionic current recorded from bullfrog sacculus hair cells in response to displacement stimuli applied at different angles with respect to the excitatory direction (0 degrees). The dashed red curve is the cosine of the stimulus angle. (Data and figure reproduced with permission from Corey and Hudspeth^42^). (**c**) The MEMS sensor output (blue line) and the cosine of the stimulus angle (black dotted line).

In order to determine the directional dependence of the sensor, the dipole was positioned at various angles with respect to the MEMS pillar bundle (Fig. 6a). The dipole was vibrated at a constant frequency and amplitude while the position of the dipole with respect to the sensor was varied between various recordings. The directionality detection experiment is conducted at a low flow velocity of 400 μm/s, which is generated by the dipole vibrating at 2 Hz. The principle of flow directionality sensing holds valid for other flow velocities as well. The amplitude fast Fourier transform (FFT) of the sensor output showed a large variation in the FFT peak as the dipole was shifted from 0° to 180° (Fig. 6a). As expected, the maximum voltage output was at 0°, because the nanofibers were stretched to the maximum extent. At 180° the sensor generated almost no output since the mechanical stress on the nanofibers is minimum. These results match well with the directional sensitivity of hair cells. For instance, the amplitude of receptor currents recorded from a patch of hair cells in the bullfrog sacculus, driven by moving the overlying otolithic membrane at different angles, varied approximately with the cosine of the angle^43^ (Fig. 6b). Figure 6c plots the sensor output as a function of stimulus angle. The results depict the similarity in directional response which further emphasizes the agreement between the sensor output and biological receptors. Imbibing the fundamental biomimetic sensing principles that offer direction-dependent sensitivity to the biological hair cell, the MEMS sensor achieves an ability to sense flow direction.

### Advantages of the biomimetic stereocilia sensor

The artificial stereocilia sensors presented in this work achieve flow velocity and flow direction sensing capability through a biomimetic design and structure. Most hair cell inspired sensors developed in the past have only presented flow velocity sensing capabilities of their sensors^24^,^25^,^28^,^30^,^31^,^33^. Some of them who performed flow direction sensing have conducted their experiments in air, but not in water^2^,^11^. Flow velocity and flow direction sensing in water is a more challenging task due to the requirement of waterproofing of the sensing elements and contacts of the sensor. In the current design, the HA-MA hydrogel encapsulation protects the PVDF tip-links and the sensor contacts from direct exposure to the external flow, which could cause electrical and mechanical damage to the sensor. In addition, the flow directionality experiment was conducted at very low flow velocity of 400 μm/s. At such low velocities most sensors fail to resolve the flow direction^32^,^44^. The sensors also achieved a threshold detection limit of 8 μm/s, which is 125 times higher than that of the sensors developed by Chen N. *et al*.^32^ and is in par with the biomimetic poly(ethyleneglycol) tetraacylate (PEG-TA) cupula sensor developed by McConney M. E. *et al*.^30^. As compared to the piezoresistive hair cell sensors developed in the past^24^,^25^,^30^,^31^,^32^,^33^, the stereocilia sensors utilize piezoelectric nanofibers as sensing elements and are thereby self-powered and do not need any external power supply to operate. The sensors are fabricated from all-polymer materials and are flexible and biocompatible as compared to hair cell sensors developed in the past^24^,^25^,^30^,^31^,^32^,^33^, allowing immense number of applications in biomedical and microfluidic devices. The fabrication approach demonstrated here can guide towards the development of other biomimetic sensors, structures and materials in the future.

## Conclusion

In general, this work demonstrates that extending the understanding of the sensing principles, materials, sensory architecture and functionality of biological hair cells can lead to novel sensors that are biocompatible, self-powered, sensitive, accurate and inexpensive. An artificial MEMS pillar bundle that mimics the structural features of the hair cell bundles in fish is designed, built, and tested. Water flow sensing experiments conducted using a dipole stimulus demonstrated a high sensitivity of 300 mV/(m/s), and a low threshold detection limit of 8 μm/s. This paper, for the first time, presents the development of a biomimetic MEMS flow sensor that resembles the hair cell sensors found in nature by mimicking their structural architecture, materials and flow sensing principle. The sensing performance of this artificial MEMS sensors closely matched that of their biological counterparts^7^,^8^,^9^. The sensor features arrays of PDMS micro-pillars with gradated height, connected with electrospun PVDF nanofibers at their tips. PVDF piezoelectric nanofibers act as biological tip-links and elicit electric charges proportional to the stress induced in the fibers. An HA-MA hydrogel cupula was developed through a drop casting process that encapsulates all the stereocilia and their nanofiber tip links. Since all the PDMS stereocilia are infused into the hydrogel cupula, when the cupula bends in response to an external flow, it causes all the stereocilia to bend as well.

The paper demonstrates how a biomimetic translation of the morphology and sensing principle of biological flow sensors resulted in a sensing performance at par with them. The biomimetic approach together with the novel fabrication methods presented in this paper will form a guide to the development of nature-inspired micro- and nano- flow sensors in the future. The sensors perform passive sensing, and thereby, bring a sea-change in the maneuvering and navigation of underwater vehicles and robots by enabling them to attain hydrodynamic artificial vision, super maneuverability and extreme control which undersea animals naturally achieve using the lateral line sensors on their body. Miniaturized size, biocompatibility, and ability to operate with no external power requirement distinguishes these sensors from the hair cell inspired MEMS sensors developed in the past and extends their applications to the field of biomedical devices and microfluidics. For example, the low velocity threshold detection achieved by the sensors will enable their usage in intravenous flow sensing applications.

Future work should involve further improvements to mimic the sensing principle and morphology of the biological hair cells to enhance flow sensitivity. Improvement of the biomimetic sensors will involve research in multidisciplinary fields, including fluid mechanics, neurophysiology, nanofabrication and soft polymer materials. For example, sensitivity can be improved by optimization of the flexibility of the PVDF tip. Another way is to include arrays of varying size artificial hair bundles encapsulated by hydrogel cupulae of different sizes. These types of morphological variations have been shown to result in 30-fold range in sensitivity^19^. Designs featuring pillar bundles that laterally stick together and pivot at the base, as is the case in their biological counterparts^13^, could further improve the sensitivity and the velocity threshold limit of the sensors. The current sensor includes one artificial bundle per cupula; a future improvement would be to include a number of artificial bundles per cupula similar to the structure of a neuromast. Simulated coupling of hair cells has been shown to reduce noise and enhance amplification of sinusoidal stimuli leading to increased sensitivity^45^.

In the biological system, flow sensitivity is ultimately dependent on the synergy of both the peripheral and the central information processing pathways^46^. Recent experiments in larval zebrafish^47^ have stimulated directly an individual neuromast and recorded from the associated afferent neuron while observing the behavioural response of the fish. Interestingly, a behavioural response was elicited by a single neuromast stimulation. Such biological studies will increase our understanding of how stimuli coding lead to behaviour^48^. With this knowledge, our ultimate goal is to put together an array of artificial neuromasts and utilize the integrated output of the sensors to optimize flow scene mapping.

## Materials and Methods

### Electrospinning

PVDF powder (MW 534000) was purchased from Sigma-Aldrich. For each electrospinning process, 1.7 g PVDF powder was dissolved in a mixture of 3.5 ml DMF (Sigma-Aldrich) and 8 ml acetone (Sigma-Aldrich) and heated at 40 °C for 120 min so that the solution became homogeneous. The resulting transparent viscous solution was transferred into a 10 ml syringe for electrospinning. A direct current (dc) voltage of 12 kV was applied across a 18 gauge syringe needle and a rotating spindle of diameter 10 cm. The polymer solution was dispensed at a feed rate of 5 μl/min. A number of pillar bundle sensors were mounted on the spindle collector which was positioned 15 cm away from the needle. The spindle rotated a speed of 1500 rpm causing the fibers to stretch as they were deposited on the PDMS pillars while being electrostatically aligned across the electrode gap.

### HA-MA hydrogel synthesis

HA powder was dissolved in de-ionized (DI) water and stirred overnight at room temperature to prepare 1.5% (w/v) solution. The following day, the HA solution was adjusted to pH 8 using 5 M NaOH while 20 molar excess MA was slowly added to it. After addition of MA, HA was allowed to react with it for 2 hr by maintaining the pH at 8. The HA-MA solution was stored at 4 °C for 24 hr. It was then dialyzed against large volume of 0.1 M NaCl solution using a dialysis bag (10 kD MWCO, 2.9-cm diameter, flat width 4.5 cm) over a magnetic stirrer at room temperature for 48 hr. The sink medium was completely replaced at intervals of 4 hr on the first day and 12 hr subsequently. After 48 hr, the HA-MA solution was dialyzed against alternating solutions of 1:4 EtOH–H_2_O (v/v) and pure H_2_O for 2 cycles. Finally, the HA-MA solution was frozen at −80 °C for 2 hr and then transferred to a freeze-drying container and lyophilized for 72 hr. After drying, the dried HA-MA fluffy powder was used to prepare 2% (w/v) solution in DI water. The HA-MA solution was stirred overnight at room temperature. The next day, 0.1% (v/v) of photoinitiator Irgacure 2959, prepared from 10% (w/v) stock solution in 70% ethanol, was added into the HA-MA-drug solution and mixed manually for a few minutes. One milliliter of this solution was then taken into a stainless steel cylindrical mould (inner diameter 1.7 cm, height 1 cm), capped at one end with parafilm, and exposed under a UV lamp (VL-8.LC, Vilber Lourmat, France) at 365 nm for 5 min. Cross-linked gel was carefully taken out of the mould and swelled in nanopure DI water for 8 hr and used for nano-indentation analysis.

### Determination of piezoelectric coefficient

To electrically characterize a single nanofiber, we fabricated a MEMS substrate that allows a single nanofiber to be suspended between the electrodes. The MEMS device consists of a deep trench etched into a silicon wafer with gold electrodes placed on both sides of the trench. During the electrospinning process, a number of MEMS devices were mounted on the rotating substrate in such a way that the fibers bridge the trench extending from one contact pad to the other. Electrospinning was conducted for an optimised time (3 s), which reduced the number of fibers deposited on the samples, and the devices with single nanofibers deposited on them were chosen for the determination of the piezoelectric coefficient. Conductive epoxy was dispensed on the two ends of the suspended fiber. The maximum deformation at the center point of the fibers was measured by applying electrical fields ranging from 0 to 1 V/μm across the electrodes and imaging the fibers under a confocal microscope (PLμ Sensorfar confocal imaging profiler). The displacements of the nanofibers observed under the confocal microscope for various electric fields applied were recorded.

### Flow-sensing experiments

In all experiments, a “dipole” stimulus was used to perform hydrodynamic characterization of the sensors under biologically relevant scenarios. As done for experiments on fluid motion detection by fish, an 8-mm sphere was vibrated using a permanent magnet mini-shaker (Brüel & Kjær model 4810, Norcross, GA; axial resonant frequency >18 kHz). The sphere was connected to the mini-shaker by a stainless steel rod 120 mm long and 2 mm in diameter, oriented along the shaker axis. The sphere was driven at a desired amplitude and frequency by a function generator amplified by a power amplifier (Brüel & Kjær model 2718). The mini-shaker was inverted and mounted on top of a water tank of dimensions 1 m (L) × 0.6 m (W) × 0.4 m (H) so that the sphere was immersed into water. The pillar bundle was mounted with pillars horizontal, so that the sphere motion was perpendicular to the pillar axis. In all the experiments, the output from the sensors was amplified 500X using a Stanford Research SRS560 low-noise pre-amplifier. The output from the sensor was digitized at 2 kHz (National Instruments, NI-DAQ) and recorded in LabVIEW. Before testing the response of the pillar bundle sensors caused by the dipole stimulus, the sphere motion was characterized in air using a Polytec PSV-300 LDV to determine the velocities generated by its vibration when driven at various amplitudes and frequencies.

## Additional Information

**How to cite this article**: Asadnia, M. *et al*. From Biological Cilia to Artificial Flow Sensors: Biomimetic Soft Polymer Nanosensors with High Sensing Performance. *Sci. Rep*. **6**, 32955; doi: 10.1038/srep32955 (2016).

## Supplementary Material

Supplementary Information

## Figures and Tables

**Figure 1:**
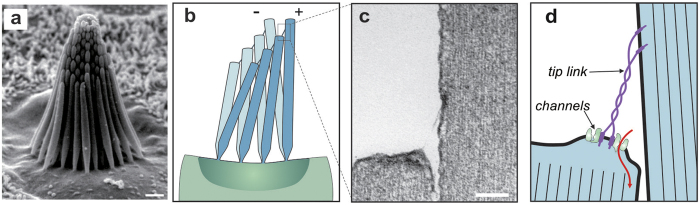
Bioinspiration: architecture and principles of mechanotransduction of the ciliary bundles in nature. (**a**) Morphology of saccular hair bundles in the bullfrog. In this organ, each bundle consists of approximately 60 stereocilia arranged into 8 rows with heights varying from 4–8 μm. (**b**) Schematic illustrating the deflection of a column of stereocilia during stimulation. Deflection of the hair bundle causes a shear between successive rows of stereocilia causing the tip links to tighten. (**c**) A transmission electron micrograph showing the tip link between two adjacent stereocilia. (**d**) Tension induced in the tip link opens ion channels. Scale bars: a = 3 μm; c = 1 μm.
