# Supplementary material for: From Biological Cilia to Artificial Flow Sensors: Biomimetic Soft Polymer Nanosensors with High Sensing Performance
Source: Sci Rep. 2016 Sep 13;6:32955. doi: 10.1038/srep32955 (PMC5020657; doi:10.1038/srep32955)
Supplement: Supplementary Information [file srep32955-s1.pdf]

From Biological Cilia to Artificial Flow Sensors: Biomimetic Soft Polymer Nanosensors with High Sensing Performance

Mohsen Asadnia[1,2+], Ajay Giri Prakash Kottapalli[3+*], K. Domenica Karavitaki[4], Majid Ebrahimi Warkiani[5] Jianmin Miao[1] , David P. Corey[4], Michael Triantafyllou[3,6]

[1]School of Mechanical & Aerospace Engineering, Nanyang Technological University,

50 Nanyang Avenue, Singapore 639798

[2]Department of Engineering, Macquarie University, Sydney, New South Wales 2109, Australia

[3]Center for Environmental Sensing and Modeling (CENSAM) IRG

Singapore-MIT Alliance for Research and Technology (SMART) Centre, 3 Science Drive 2, Singapore 117543

[4]Howard Hughes Medical Institute and Department of Neurobiology, Harvard Medical School, 220 Longwood Avenue, Boston, MA 02115, USA

[5]School of Mechanical and Manufacturing Engineering, Australian Centre for NanoMedicine,

University of New South Wales, Sydney, New South Wales 2052, Australia

[6]Department of Mechanical Engineering, Massachusetts Institute of Technology,

77 Massachusetts Avenue, Cambridge, MA 02139, USA

[*]Corresponding author (Email): ajay_k@mit.edu, (Tel): +65 67906038, (Fax): +65 67906995

Supplementary information S1

Nanofabrication of artificial ciliary bundle

The fabrication of the proposed artificial hair cell flow sensor combines soft-polymer synthesis methods and nanofiber generation techniques with conventional microfabrication methods to form novel flow sensors. The sensor fabrication consists of three major steps: (1) fabrication of artificial stereovilli - pillars with varying heights; (2) formation of artificial tip-links - electrospun PVDF nanofiber sensing elements; (3) development of biomimetic cupula - HA-MA hydrogel cupula.

Initially, pillars with graded heights were developed through microfabrication using SU-8 negative photoresist. Later on, through double moulding of PDMS, the pillar bundles were transferred to form flexible PDMS pillars. In order to form the desired gradient in SU-8, we conducted partial development which utilizes the low development rates at the convex corners under no agitation conditions. Figure 1 shows the fabrication process to form the SU-8 stereocilia bundle with height gradient. Initially, a glass wafer of 300 μm thickness was photopatterned with gold (Au) patterns through lift-off process. For this purpose, firstly, the glass wafer was treated with ion-exposure in a hexamethyldisilazane (HMDS) chamber to make the surface hydrophilic. A 5 μm thick AZ9260 photoresist was then spin-coated on the

glass wafer. A bright-field glass mask with circular chrome patterns of 50 μm diameter was used to pattern the photoresist. Thereafter, a 500 nm Au layer was deposited on the glass wafer with a 20 nm Ti adhesion layer. The glass wafer was then dipped into acetone for 2 hours to lift-off photoresist and to form the desired gold pattern (Fig. 1a). The glass wafer acts as a substrate as well as the mask for photo-patterning the SU-8 pillar structures.

The next step is to form the SU-8 pillars with height gradient. Before spin coating, the glass wafer was baked on a hot plate at 110 ^{0}C for 5 min to remove any residual moisture on the wafer surface and enhance the adhesion of SU-8 layer on the glass surface. This step was followed by HMDS exposure of the glass wafer on the surface to be coated with SU-8. The glass wafer was turned upside down and was spin-coated with a thick layer of SU-8 2150 (on the non-patterned side) as shown in Fig. 1b. 10 ml of SU-8 2150 was dispensed and allowed to spread due to gravity for about 5 min. Spin-coating was done at 500 rpm for 10 s with an acceleration of 100 rpm/s and followed by 900 rpm for 30 s with an acceleration of 300 rpm/s. After spin-coating, the wafer was transferred to a hotplate for the prebaking process conducted first at 65 °C for 30 min and then slowly ramped up to 95 °C where it was maintained for 150 min. Inadequate prebaking of the SU-8 layer could cause the SU-8 to stick to the mask during the lithography process. Therefore, the baking processes were conducted for prolonged times with slow temperature ramping to avoid inbuilt stresses in the thick SU-8 layer which will affect the shape of the pillars after the exposure and developing process.

Before the circular patterned side of the glass wafer was used to expose the SU-8, it was necessary to create rectangular blocks of exposed (hardened) regions of SU-8, which formed the convex regions generating the required height gradient.

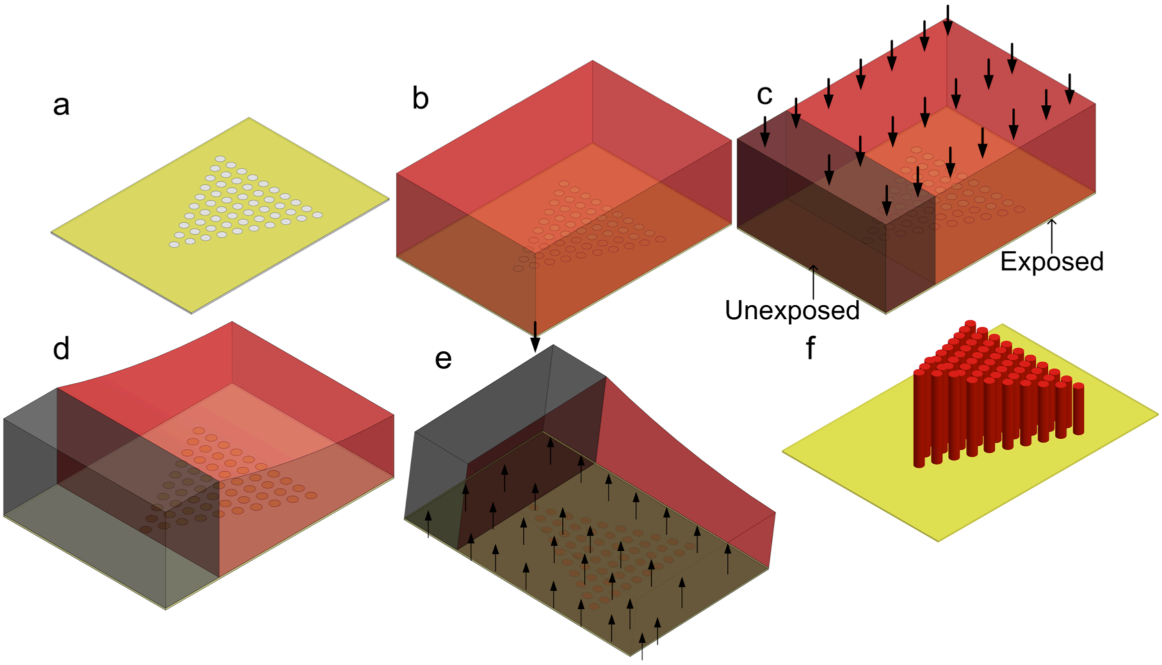

Figure 1 | Fabrication of graded-height PDMS pillars: (a) Lift-off process to form Au circular patterns at the backside of a glass wafer. **(b)** A 810 μm thick SU-8 layer of uniform thickness. **(c)** Exposed (hardened) region of SU-8 created at the edge of the tallest pillar to generate a gradient of SU-8 needed to form pillars of graded heights. **(d)** A gradient in SU-8 formed through partial time-controlled developing process. **(e)** Exposure from the backside of the glass wafer through Au patterns to cross-link SU-8 and form the pillars. **(f)** Final developing process to form the pillars of graded heights.

The SU-8 layer was exposed through a dark-field glass mask with rectangular openings that were aligned and positioned at the edge of the tallest pillar. The SU-8 layer was then exposed for 72.6 s using a MA6 Karl Suss mask aligner machine (Fig. 1c). The SU-8 layer was post-baked at 65 °C for 15 min, and then the hotplate was ramped up to 95 °C and maintained there for 40 min. Thereafter, the wafer was immersed in SU-8 2150 commercial developer for 12 min (Fig. 1d). The developing time is the most crucial step in forming the appropriate graded-height pillars. Low developing times lead to the formation of pillars with a small height. Over-developing causes the step heights too close to 90^0 and thus, most of the shorter pillars (those away from the hardened SU-8 block) will be completely developed and vanished. Surface profiler and SEM were used to verify the gradient formed in the SU-8 for various developing times. This process was followed by another soft-baking process for the second exposure of the wafer through circular Au patterns. This second soft-baking process was conducted at reduced temperatures since the SU-8 layer already underwent a soft- and post-baking processes. The soft-baking was followed by 89 s of SU-8 exposure through the Au patterned side of the wafer (Fig. 1e). During this step, there is some diffraction of UV

light through the thickness of the glass wafer since the Au pattern is on the other side of the glass wafer leading to pillars with wider base. The formation of pillars with wider base diameter adds an advantage to the process for two main reasons. First, SU-8 processes that perform top exposure would result in pillar structures with a "mushroom top" or "T-top", which would risk pillar uprooting during the PDMS molding process. Second, the reverse "T-top" formed using our fabrication method leads to pillars with a larger diameter at the base enabling the sensors to survive high flow velocities. After the exposure the wafer was post-baked at reduced temperatures. This step was followed by SU-8 development process without agitation for 30 min (Fig. 1f). As the developing process proceeds, the pillars get exposed to the developing solution and agitation could cause bending of the pillars. The wafer was then baked on a hotplate for 1 hr at 50 ^{0}C. After forming the SU-8 graded-height pillars, the entire structure was transferred to PDMS by performing double moulding on the glass wafer with SU-8 pillars. Initially the SU-8 pillars were made hydrophilic by performing an oxygen plasma surface treatment for 90s. The wafer was then exposed to silane for two hr under vacuum to provide a super hydrophobic surface. Due to the hydrophilicity of the surface (due to the oxygen plasma treatment), silane uniformly forms a thin super-hydrophobic layer all over the SU-8 pillar bundle. This helps to remove the PDMS mould without breaking the SU-8 pillars. The degassed PDMS mixture was then poured uniformly all over the wafer template with SU-8 pillars. The template was baked in an oven at 80^{0}C for 45 min to allow the PDMS to cure. The PDMS was then carefully peeled off from the wafer and was used as the main mould to make the graded height pillars. Figure 2a shows an SEM image of the SU-8 pillars. Figure 2b-c show the SEM and optical images of the arrays of 55 PDMS pillars with varying heights that are formed after the microfabrication and double moulding process.

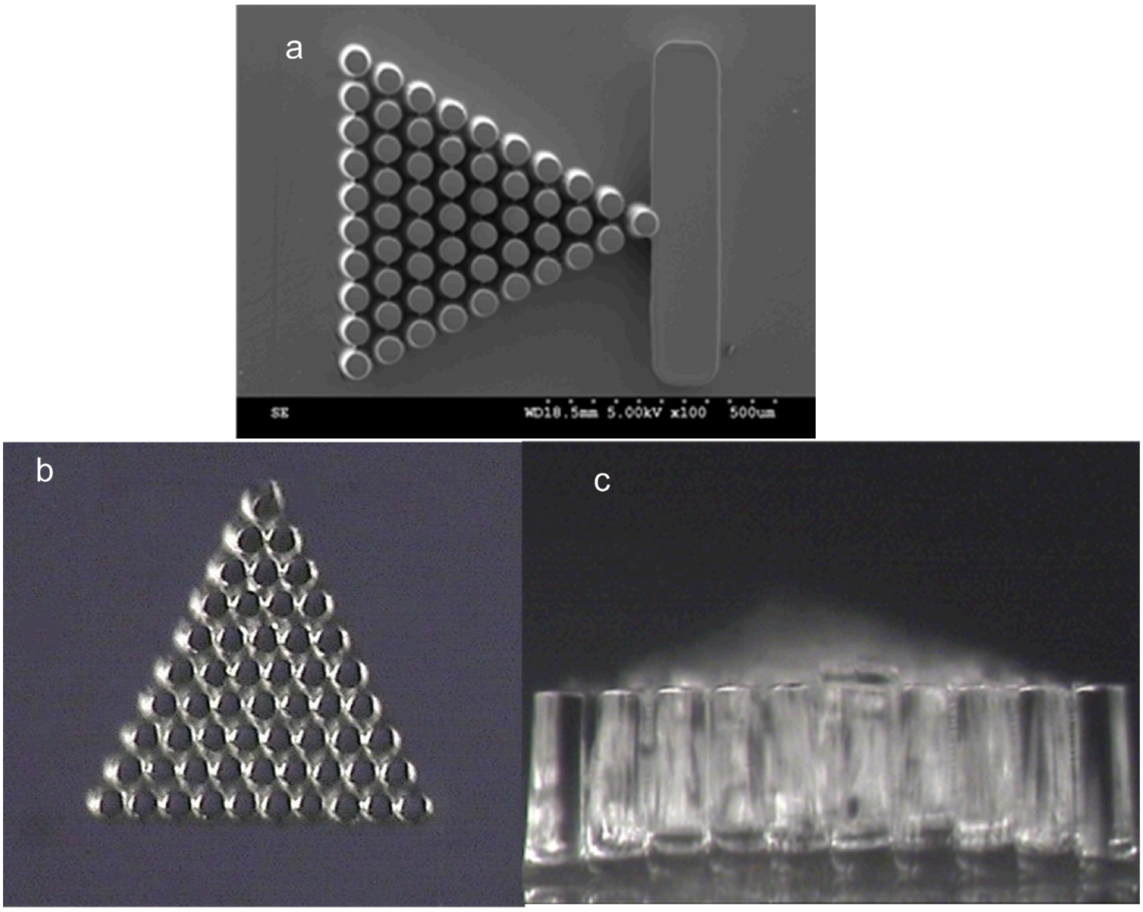

Figure 2 | Artificial PDMS hair cell: (a) SEM image of SU-8 pillars with height gradient. **(b)** Top view showing the array of 55 PDMS pillars taken using optical microscope. **(c)** Angle-view showing the height gradient formed after the entire fabrication (In (c) Note that the height variation occurs perpendicular to the plain of the paper).

Supplementary information S2

Nanofiber artificial tip-links

Analogous to the biological tip-links, we developed artificial PVDF nanofiber tip-links through electrospinning process to connect the distal tips of the pillars. PVDF is a semi-crystalline polymer with a structure consisting of linear chains with hydrogen and fluoride in sequence around a carbon backbone with a simple chemical formula (CH_2–CF_2). The chemical structure of PVDF falls between the structure of Polytetrafluoroethylene (PTFE; CF_2-CF_2) and Ethylene (CH_2-CH_2). The structural similarity to ethylene provides PVDF with a great flexibility and the crystalline similarity with PTFE gives PVDF a stereochemical constraint.

In this study, we used the far field electrospinning (FFE) process with a rotating collector to achieve aligned PVDF nanofibers. We conducted optimization of various electrospinning parameters to achieve PVDF nanofibers that demonstrate high β-phase with the minimum amount of beads. An SEM image of the aligned PVDF nanofibers is shown in Fig. 3a. The average diameter of the nanofibers achieved was 870 nm (shown in insert). Through careful optimization of the electrospinning process we have also succeeded in obtaining single fibers on various substrates (Fig. 3b). Obtaining single fiber between desired electrodes is necessary to characterize the fiber's mechanical and piezoelectric properties. This section presents the material and mechanical characterizations conducted on the electrospun PVDF nanofibers. The electrical characterization to determine the piezoelectric coefficient is explained in the main manuscript.

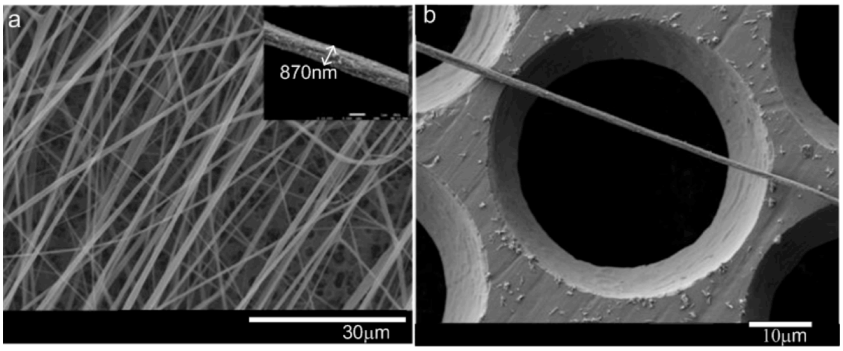

Figure 3 | Electrospinning process optimization: (a) Aligned nanofibers formed after optimization of flow rate of the solution and spinning speed of the rotating disk collector. Inset shows a zoom-in view showing a single nanofiber. **(b)** A single fiber on a TEM grid used to study the mechanical characteristic of a single PVDF nanofiber.

Material characterization of PVDF nanofibers

Following the optimization step, we performed x-ray diffraction (XRD) and Fourier transform infrared (FTIR) spectroscopy to observe the material structure and determine the crystalline structure of the material. The XRD patterns of the PVDF nanofibers were observed with a Siemens D5000 X-ray diffractometer with Cu Kα radiation (λ = 1.54 Å) (Fig. 4a). The tests were conducted in reflection mode at ambient temperature with two theta (degree) varying between 10° and 50°. The scanning speed and the step size used were 1 min^{-1} and 0.02°, respectively. XRD (Fig. 4a) revealed a strong peak at 20.6° showing a dominant β-phase structure.

In order to obtain the FTIR spectra of the nanofibers, the samples were placed on top of the attenuated total reflectance (ATR) set and scanned from 600 to 1500 cm^{-1}. A total of 32

scans were collected for signal averaging. The ferroelectric all-trans β-phase has characteristic absorption bands at 840 cm^{-1} (Ch_2 rocking), 1073 cm^{-1} (C-C stretching band), 1275 cm^{-1} (Trans band) and 1430 cm^{-1} (CH_2 band) are indexed (Fig. 4b).

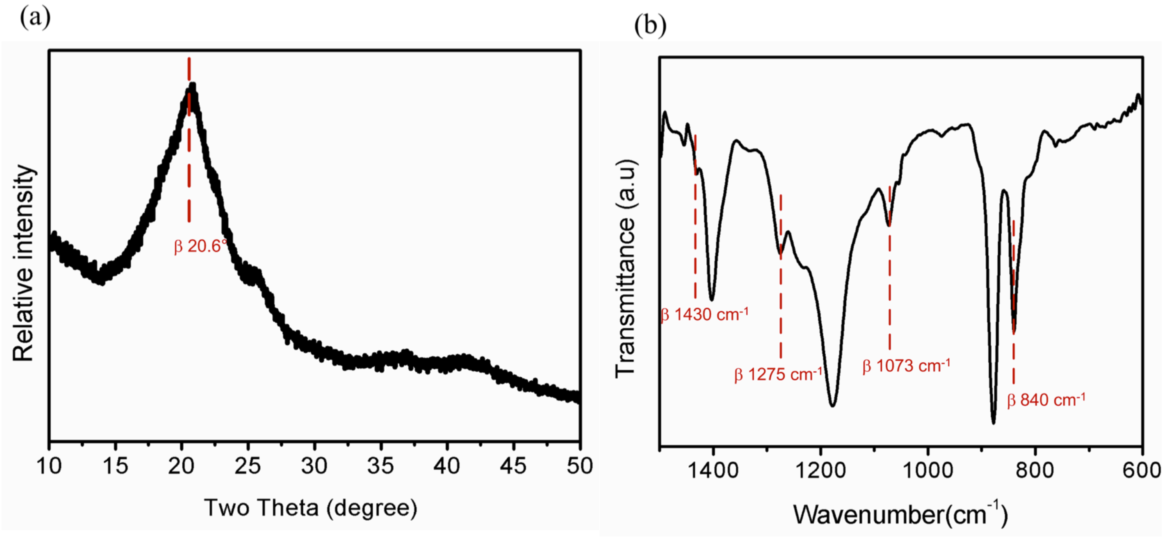

Figure 4 | Material characterization of PVDF fibers (a) XRD pattern of electrospun PVDF nanofiber. **(b)** FTIR spectra in the ATR mode where β-phases are labeled.

Mechanical characterization of PVDF nanofibers

We have conducted mechanical characterization of a single nanofiber through nano-indentation analysis to investigate the Young's modulus and hardness of a single fiber. The mechanical properties of the fibers are probed using a TriboScan 950 (Hysitron, MN, USA). The machine is equipped with a cube corner tip and the test is done in scanning probe microscope (SPM) mode. Due to the sample size, a 50 μN load with 5 s, 2 s and 5 s (loading-holding-unloading) load function was applied on the samples in the piezo automation mode. Indentation points were chosen at the apex of the fibers. The cube corner tip was calibrated with a standard fused quartz sample for 100– 400 nm contact depths. The nano-indentation studies of the fibers revealed that the elastic modulus and hardness of the samples are 2.2 GPa and 0.1 GPa, respectively (Fig. 5).

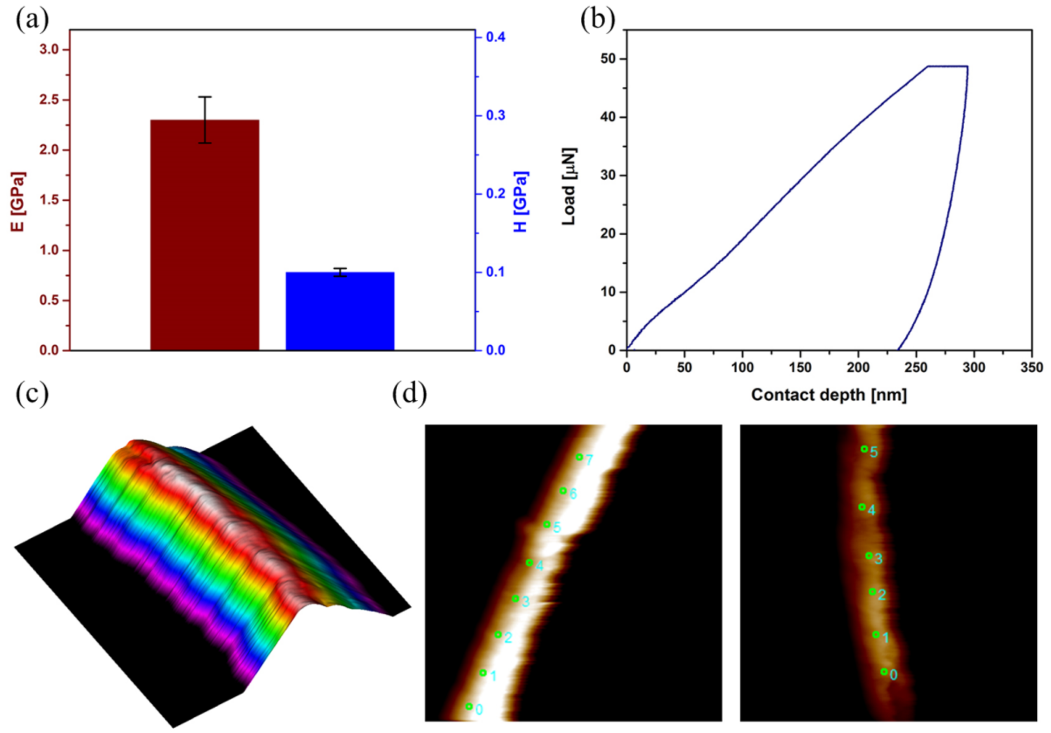

Figure 5 | Mechanical characterization of the nanofibers. (a,b) The nanoindentation studies of the fibers revealed that the elastic modulus and hardness of the samples are 2.2 GPa and 0.1 GPa, respectively. Due to the sample size, a 2D transducer equipped with a cube corner tip was used for imaging and probing the mechanical properties of the sample in scanning probe microscope (SPM) mode. **(c,d)** SPM images presented the 3D topography and position of indents on the fibers.

Supplementary information S3

PVDF nanofiber placement on PDMS pillars

The following steps describe the electrospinning process conducted on the polymer pillar bundle to form the tip-links.

First, individual pillar bundles were diced out of the flexible PDMS substrate. The devices were visualized under an optical microscope to precisely position very thin layers of conductive epoxy (EPO TEK H20E) along the shortest and the tallest edge of the pillars. The conductive epoxy contact pads will acquire electric charges resulting from the stretching of the nanofibers caused by the displacement of the pillars, which will be recorded as sensor output. To place the nanofibers on top of the pillars, first a number of devices were simultaneously mounted on the rotating disc collector of the electrospinning machine. Far-field spinning was then performed to form the aligned PVDF nanofibers. The disc was rotated at a uniform speed of 1500 rpm (that was pre-optimized to form nanofibers with high piezoelectric coefficient). This ensures that the desired mechanical stretch was generated on

the fibers resulting in high piezoelectricity. PVDF nanofibers connecting the pillars parallel to the axis of the height gradient were formed due to the uniform rotation of the mandrel.

Supplementary information S4

Artificial HA-MA hydrogel cupula

The final step in the fabrication of the artificial hair cell flow sensor was to encapsulate the pillars and nanofibers inside a structure that mimics the cupula. 2 % (w/v) and 4 % (w/v) Hyaluronic acid methacrylic anhydride (HA-MA) hydrogels with 0.1 % Irgacure 2959 initiator concentrations were chosen as suitable precursors since they provided a close match of the Young's modulus to the biological cupula[38]. The Young's modulus of the hydrogel sample was verified by performing nanoindentation and rheology. A G200 Agilent Technologies nano-indenter machine was used to determine the hardness and Young's modulus of the artificial hydrogel cupula. Dynamic oscillatory shear tests were conducted using stress-controlled rheometer (Physica MCR 501, Anton parr, USA).

For the rheology and nanoindentation experiments, the HA-MA hydrogel material was moulded into flat discs of 25 mm diameter. The nanoindentation was performed on the flat face of the moulded hydrogel using a cono-spherical tip. Nanoindentation studies showed that the hydrogel samples had a Young's modulus of 8-10 Pa. Figure 6*a* shows the results of 5 indentations performed at five locations of the HA-MA sample surface. Rheology was used to characterize the mechanical properties of the hydrogel. The shear modulus G' (both storage and loss modulus) and the complex viscosity were measured at various frequencies. Figure 6*b* shows the change in elastic modulus of the hydrogel as a function of frequency. Even with high-applied strain there is small change in the modulus, indicating minor time-dependence in the mechanical response of the sample. Rheological measurements estimated a Young's modulus of 120 Pa. The loss modulus follows a similar trend but has a smaller Young's modulus of 30-100 Pa. The complex viscosity decreases linearly with frequency as seen in Fig. 6*c*.

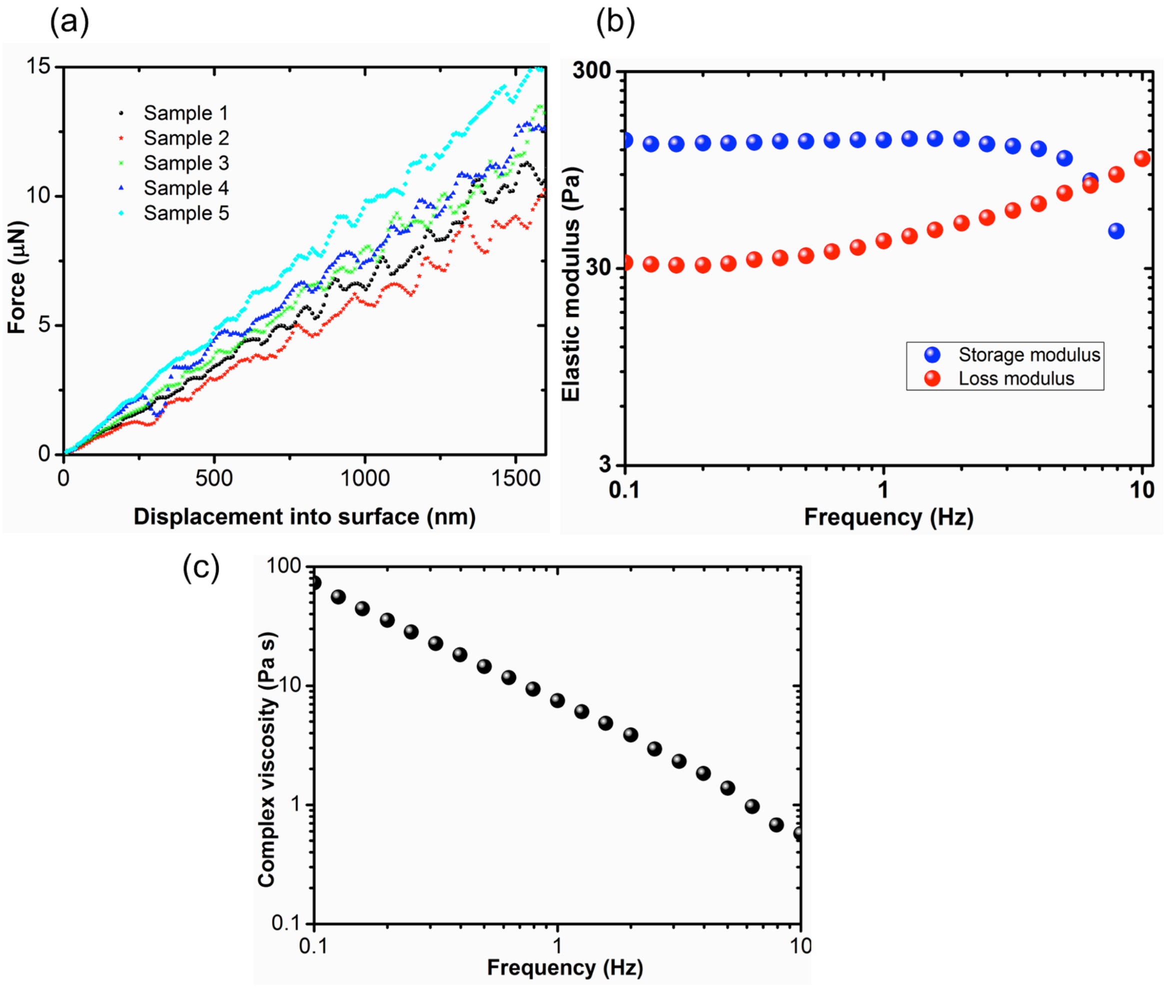

Figure 6 | Material characterization of the HA-MA hydrogel cupula (a) Nanoindentation results from 5 runs. **(b)** Storage and loss modulus as a function of frequency **(c)** Complex viscosity as a function of frequency.

PVDF nanofibers generated by FFE process are super hydrophobic and do not let the hydrogel seep through the tip links and reach the base of the bundle. Moreover, the air trapped between the narrow gaps of the PDMS pillars also leads to a superhydrophobic bundle that resist the hydrogel from seeping through. Therefore, an oxygen plasma treatment for 90 s was performed prior to hydrogel drop-casting to modify the surface properties of the nanofibers and make them superhydrophilic. Our experiment shows the contact angles formed by sessile liquid drops on a PVDF electrospun nanofiber substrate before and surface treatment were 162.8° and 10.7°, respectively.

Hydrogel drop-casting was performed in a two-step process. Initially a 2% hydrogel solution of was drop-casted followed by a 4% hydrogel solution. The 2% hydrogel solution was less viscous and flowed through the nanofibers and the gaps between the pillars and

binds them together. The second step of drop-casting 4% hydrogel was to ensure that the hydrogel cupula had a desired high-aspect ratio and did not spread over onto the contact pads.

References

[38] McHenry, M. J. & van Netten, S. M. The flexural stiffness of superficial neuromasts in the zebrafish (Danio rerio) lateral line. *J. Exp. Biol.* **210**, 4244-4253; DOI:10.1242/jeb.009290 (2007)
